# Maca polysaccharides: structural diversity, biological activities, and structure–activity relationships

**DOI:** 10.1007/s13659-026-00609-z

**Published:** 2026-07-06

**Authors:** Xue-Fang Zhao, Zhou-Wei Wu, Xiao-Qiu Liu, Ming-Hua Qiu

**Affiliations:** 1 School of Traditional Chinese Medicine, Shenyang Pharmaceutical University, 103 Wenhua Road, Shenyang District, Shenyang, 110016 Liaoning People’s Republic of China; 2 State Key Laboratory of Phytochemistry and Natural Medicines, Kunming Institute of Botany, Chinese Academy of Sciences, Kunming, 650201 Yunnan People’s Republic of China; 3 University of Chinese Academy of Sciences, Beijing, 100049 People’s Republic of China

**Keywords:** Maca polysaccharides, Structural characterization, Biological activity, Structure–activity relationship

## Abstract

Maca (*Lepidium meyenii* Walp.) is a high-altitude cruciferous crop native to the Peruvian Andes. Its edible hypocotyl accumulates structurally diverse polysaccharides that exhibit a broad spectrum of bioactivities, including immunomodulatory, antioxidant, anti-fatigue, and hepatoprotective effects. This review systematically summarizes recent advances in the extraction, purification, and structural characterization of maca polysaccharides. Five major structural types have been identified: α-glucans, arabinogalactans (types I and II), homogalacturonans, rhamnogalacturonans, and mannose-containing heteropolysaccharides, with molecular weights spanning three orders of magnitude (3.0–1951.0 kDa). We critically analyze structure–activity relationships, demonstrating that biological functions are governed by the interplay of molecular weight, monosaccharide composition, glycosidic linkage patterns, and spatial conformation. Key findings reveal that medium-molecular-weight fractions (200.0–400.0 kDa) exhibit optimal immunomodulatory activity, specific monosaccharide motifs confer receptor selectivity, and triple-helix conformations enhance immune recognition. Current limitations—including structural heterogeneity, incomplete mechanistic understanding, and lack of clinical validation—are discussed, alongside future research priorities. This work provides a comprehensive framework for understanding maca polysaccharide structure–function relationships and guiding their future development as functional food ingredients.

## Introduction

Maca (*Lepidium meyenii* Walp.), a cruciferous crop native to the central Andes of Peru, is mainly cultivated at altitudes of approximately 3,500–4,500 m above sea level [1]. The extreme high-altitude environment is associated with the distinctive physiological adaptations of maca and its high accumulation of diverse bioactive constituents. For more than 2,000 years, indigenous people in the central Andes have consumed maca as both a staple food and a traditional remedy, mainly to enhance physical strength and fertility and to relieve fatigue [2]. In recent decades, maca cultivation has expanded beyond its native range to other high-altitude regions worldwide. In China, maca was approved by the Ministry of Health in 2002 and has since been cultivated in Yunnan, Xinjiang, and Tibet; the first successful cultivation was in Lijiang, Yunnan Province [3]. In 2011, maca powder was officially registered as a novel food resource in China [4]. India has also emerged as a cultivation region where suitable climatic conditions exist [5]. Peru remains the leading producer, while major consumer countries include the United States, Canada, United Kingdom, Germany, China, Japan, and the Netherlands [6]. With the growing global demand, maca has increasingly been developed as a functional food ingredient. Among its multiple bioactive fractions, polysaccharides have become one of the major focuses of current research [7, 8].

Modern research shows that maca is rich in essential nutrients, including protein (9.56–21.90%), carbohydrates (46.1–74.8%), and fats (0.59–2.20%) [4]. Beyond these basic nutrients, maca also contains a variety of bioactive components, including macaenes, macamides, glucosinolates, phytosterols, alkaloids, as well as non-starch polysaccharides and immunomodulatory proteins [4, 9]. Among these bioactive components, macamides and macaenes are recognized as characteristic constituents of maca, and traditional use and modern pharmacological studies have demonstrated their contribution to the beneficial effects on sexual function and fertility [10, 11]. Collectively, these nutrients and bioactive constituents are considered to underpin the reported improvements in physical performance and reproductive function observed in preclinical and clinical studies.

Among the various bioactive constituents of maca, polysaccharides constitute one of the major macromolecular fractions and have attracted increasing research interest in recent years. Proximate analyses indicate that carbohydrates account for approximately 60–75% of the dry weight of maca. After enzymatic removal of starch, the remaining total polysaccharides typically exceed 10% of the dry matter, highlighting the prominent contribution of non-starch polysaccharides to its carbohydrate fraction [12, 13]. Maca polysaccharides exhibit pronounced immunomodulatory effects, activating macrophages, stimulating the production of mediators such as nitric oxide (NO) and cytokines including tumor necrosis factor-alpha (TNF-α) and interleukin-6 (IL-6), and enhancing phagocytic activity both in vitro and in vivo [14, 15]. Systematic investigations of maca polysaccharides started relatively late compared with low-molecular-weight constituents such as macamides and macaenes. Nevertheless, advances in chromatographic separation and purification techniques have enabled the isolation of highly purified, structurally defined fractions and have substantially advanced structure–function studies of these polymers. Accumulating structural and pharmacological studies have demonstrated that maca polysaccharides comprise a complex mixture of distinct structural types and display a broad spectrum of biological activities. Structurally, maca polysaccharides span a broad molecular-weight range, from low-molecular-weight fractions of approximately 6.7 kDa to ultra-high-molecular-weight α-glucans of around 1.9 × 10^3^ kDa [16, 17]. They also differ markedly in monosaccharide composition—predominantly glucose, galactose, arabinose and mannose, with varying amounts of uronic acids and rhamnose—as well as in glycosidic linkage patterns, including α-(1 → 4), α-(1 → 6) and β-(1 → 3) motifs. Functionally, maca polysaccharides have been shown to exert antifatigue, immunomodulatory and antioxidant effects, and they have also been reported to influence gut microbiota composition in in vitro fermentation systems and emerging in vivo models [17, 18]. Increasing evidence further suggests that these bioactivities are tightly associated with key structural parameters such as molecular-weight distribution, monosaccharide composition and glycosidic linkage patterns [19].

Despite the growing number of studies on maca polysaccharides, dedicated reviews that systematically correlate well-defined structural features with specific biological activities remain scarce. Recent summaries still emphasize that the structure–activity relationships of these polysaccharides are largely unresolved [5]. Most available studies either isolate and characterize individual maca polysaccharide fractions or assess one or a few biological activities in specific in vitro or in vivo models, rather than integrating structural information and multi-dimensional bioactivity profiles. A few studies have begun to compare fractions differing in molecular weight, monosaccharide composition or branching patterns. Nevertheless, our overall understanding of how specific structural parameters—such as molecular-weight distribution, sugar composition and glycosidic linkages—govern the diverse biological activities of maca polysaccharides remains fragmentary.

In line with the situation observed for many plant polysaccharides, current research on maca polysaccharides still faces several methodological and translational challenges [16]. First, the complexity and diversity of their structures make it difficult to obtain well-defined, homogeneous components. Second, the effects of different extraction and purification methods on the structure and activity of maca polysaccharides are not yet fully understood. Furthermore, the molecular mechanisms behind their biological activities need to be explored in greater depth. Finally, there is a significant gap between laboratory research and industrial application. This review systematically summarizes recent advances in the extraction, purification, structural characterization, and biological activities of maca polysaccharides, with particular emphasis on elucidating structure–activity relationships.

## Development of extraction and purification techniques for maca polysaccharides

### Optimization and improvement of traditional extraction methods

Hot water extraction is a classical method for isolating plant polysaccharides and takes advantage of their good solubility in hot water [20]. The extraction of maca polysaccharides has progressively evolved from simple crude procedures to more refined, optimized processes. Early work generally relied on simple boiling under basic conditions. The polysaccharide yields varied considerably depending on extraction parameters, with initial crude extractions often achieving relatively modest yields [17]. With a better understanding of process parameters, researchers began to systematically optimize key factors—such as extraction temperature, time, liquid-to-solid ratio and pH—using statistical tools including orthogonal designs and response surface methodology [21].

Systematic optimization studies using response surface methodology have established that hot water extraction of maca polysaccharides performs optimally at 77–100 °C, liquid-to-solid ratios of 20:1–40:1, and extraction times of 2–3 h, achieving yields of 10–46% depending on maca source and process conditions [17, 22]. This method is advantageous for its simplicity, safety, low cost, and suitability for large-scale industrial production. However, hot water extraction has significant limitations. High temperatures and prolonged extraction times can cause molecular chain breakage of heat-sensitive polysaccharides, reduce their molecular weight, and alter their spatial conformation, ultimately affecting biological activity [23]. Studies suggest that extraction under milder conditions may better preserve the immune activity of maca polysaccharides. For example, ultrasound-assisted extraction at 50 °C yielded polysaccharides with superior immunoregulatory activity compared to conventional hot water extraction [24].

To address these limitations, various modern extraction techniques have been developed. Additionally, pretreatment steps such as degreasing with petroleum ether and decolorization with ethanol are commonly employed to reduce impurity interference prior to extraction.

### Application and development of modern extraction techniques

Ultrasound-assisted extraction (UAE) is an efficient and eco-friendly technique that has been widely applied for polysaccharide extraction in recent years [25]. The principle involves utilizing physical effects such as cavitation, mechanical vibration, and thermal effects of ultrasonic waves to disrupt plant cell walls and accelerate the release and diffusion of intracellular substances [26]. Studies have shown that under optimized conditions (50 °C, 150 W/L power intensity, 15 min extraction time, 1:10 material-to-liquid ratio, 20/35 kHz alternation dual-frequency mode), ultrasound-assisted extraction increased the yield of purified polysaccharides by 44.90% compared to conventional hot water extraction [24].

Beyond improving extraction efficiency, UAE offers the critical advantage of milder processing conditions. Compared with traditional hot water extraction requiring several hours at elevated temperatures, ultrasound-assisted extraction can be completed within 15 min at 50 °C, significantly shortening extraction time and lowering extraction temperature [27]. These mild extraction conditions help minimize thermal degradation of polysaccharide molecular chains, preserve the integrity of glycosidic bonds, and maintain the original spatial conformation and biological activity of polysaccharides [23]. Infrared spectroscopy analysis confirmed that the key characteristic peak positions of ultrasound-extracted maca polysaccharide (US1) were essentially identical to those of hot water-extracted polysaccharide (RS1) at 3226, 2926, and 1613^cm-1^, indicating that ultrasound treatment did not alter the basic structural characteristics of the polysaccharide [24]. However, precise control of ultrasonic power is crucial. Insufficient power may fail to generate adequate cavitation for efficient extraction, while excessive power can cause mechanical breakage and degradation of polysaccharide molecular chains, ultimately reducing extraction yield. Therefore, optimizing ultrasonic parameters—particularly the precise matching of power density, frequency combination, and pulse time—is essential for successful UAE application.

Microwave-assisted extraction (MAE) takes advantage of the rapid and selective heating of microwaves to increase the internal temperature of plant cells, generating internal pressure that leads to cell rupture [28, 29]. The most significant feature of MAE is its extremely short extraction time, typically ranging from a few minutes to half an hour depending on the plant matrix [30]. While MAE has been successfully applied to extract other bioactive compounds from maca, such as glucosinolates using combined ultrasonic-microwave extraction (optimal conditions: 470 W, 70 s, achieving 0.51% glucosinolate content), its application to maca polysaccharide isolation remains to be systematically investigated [31]. Despite the short extraction time, MAE may cause uneven heating. Local overheating can induce thermal degradation of polysaccharides, as demonstrated in studies on other plant polysaccharides [32].

Enzyme-assisted extraction (EAE) promotes the release of plant polysaccharides by selectively degrading cell wall components using specific enzymes [33]. In a study comparing extraction methods for maca polysaccharides, maca was treated with 1% (v/w) Shearzyme Plus enzyme in acetate buffer (pH 4.5) at 50 °C for 12 h [34]. Enzyme pretreatment yielded lower polysaccharide recovery compared to hot water extraction, but produced extracts with reduced protein contamination. The results showed that enzyme pretreatment yielded 6.22% maca polysaccharides (MRP-E), which was lower than hot water extraction (13.08%). The protein content of MRP-E was only 1.71%, significantly lower than 7.71% for the ultrasonic-assisted extraction sample, suggesting that enzymatic treatment effectively reduced protein contamination in the extracts. Generally, EAE offers mild reaction conditions that may help preserve polysaccharide molecular structures. Additionally, the specificity of enzymatic action can facilitate the removal of certain impurities. However, the high cost of enzyme preparations and the strict requirements of process conditions have limited the application of this technology in the industrial production of maca polysaccharides.

### Innovative development of green extraction technology

In recent years, the increasing emphasis on environmental protection and sustainable development has driven the advancement of green extraction technologies. Ultrasound-assisted extraction based on deep eutectic solvents (DES) represents this trend [35]. DES are low-eutectic mixtures formed by hydrogen bonding between two or more components, characterized by low toxicity, good biodegradability, and straightforward preparation.

In maca polysaccharide extraction, choline chloride-urea DES (1:3 molar ratio) combined with ultrasound-assisted extraction demonstrates significant advantages [36]. Under optimal conditions determined by Taguchi experimental design (DES water content 30%, ultrasonic power 300 W, extraction time 20 min), the extraction yield of maca polysaccharides reached 26.28%, approximately 117% higher than water-based ultrasound-assisted extraction (12.1%). The high extraction efficiency of DES is attributed to its unique physicochemical properties: the strong hydrogen-bond network enhances polysaccharide solubility, low surface tension facilitates penetration of plant cell walls, and tunable polarity enables selective extraction of target components.

Three-phase partitioning (TPP) is another emerging green separation technique. This method establishes a three-phase distribution system by adding specific salts (e.g., ammonium sulfate) and organic solvents (e.g., tert-butanol) to the extract, enabling selective separation of polysaccharides [37]. TPP has been successfully applied to fungal polysaccharide extraction. For instance, exopolysaccharides from Phellinus baumii were extracted with 52.09% yield under optimal conditions (20% w/v ammonium sulfate, cultured broth to tert-butanol ratio of 1:1.5 v/v, 35 °C, 30 min), with polysaccharides distributed mainly in the lower aqueous phase [37]. However, the application of TPP to maca polysaccharide extraction remains to be investigated. Compared with traditional ethanol precipitation, TPP reduces the consumption of large amounts of ethanol and minimizes environmental impact from organic solvent use.

### Systematic development of separation and purification techniques

Maca polysaccharides show significant structural heterogeneity, which poses special requirements for their separation and purification. Studies have revealed that maca polysaccharides span nearly three orders of magnitude in molecular weight, from low-molecular-weight glucan (3.1 kDa) to high-molecular-weight α-glucan (1951.0 kDa). These polysaccharides comprise neutral sugars (glucose, galactose, arabinose) and acidic sugars (galacturonic acid), forming diverse structural types including glucan, arabinogalactan, and pectin polysaccharides [38, 39]. This structural diversity necessitates the adoption of fractionation strategies to obtain well-defined polysaccharide components for structure–function studies.

Graded alcohol precipitation serves as the primary approach for initial separation of maca polysaccharides. This method exploits differences in polysaccharide solubility in ethanol–water mixtures, which varies according to molecular weight, structure, and polarity. Its principle is based on the differences in solubility of polysaccharides with different molecular weights and polarities in ethanol water mixed solvents. Using ethanol concentration gradients of 40%, 50%, 60%, 70%, and 80%, five polysaccharide fractions (MP1-MP5) were successively precipitated from maca aqueous extract [40]. The effectiveness of gradient separation correlates with maca polysaccharide structural characteristics: high-molecular-weight α-glucans precipitate at lower ethanol concentrations, while low-molecular-weight, carboxyl-rich pectin polysaccharides require higher ethanol concentrations for complete precipitation. The alcohol precipitation process should be performed at 4 °C to prevent microbial contamination, followed by thorough dialysis to remove residual ethanol and low-molecular-weight impurities.

Ion-exchange and gel filtration chromatography are essential techniques for obtaining high-purity maca polysaccharides. Diethylaminoethyl (DEAE)-52 or DEAE-Sepharose columns, positively charged under neutral to weakly alkaline conditions, selectively adsorb acidic polysaccharides containing galacturonic or glucuronic acid, while neutral polysaccharides elute unretained. Acidic components with varying charge densities can be further fractionated using 0.1–1.0 M NaCl gradient elution. A two-step purification strategy combining DEAE-52 ion-exchange and Sephacryl S-500 gel filtration chromatography yielded maca polysaccharide MP21 (368.0 kDa) with high homogeneity [41]. Gel filtration chromatography (Sephadex G-50, G-100, or G-200) enables desalting and molecular weight fractionation based on size exclusion principles. Two purified fractions, MPS-1 (7.6 kDa) and MPS-2 (6.7 kDa), exhibited single symmetric peaks on high-performance liquid chromatography (HPLC) analysis, confirming high molecular weight homogeneity [42].

The selection of purification strategies should be guided by research objectives. Structural characterization typically requires high-purity samples, achievable through a three-stage purification process comprising alcohol precipitation, ion-exchange chromatography, and gel filtration. This complete purification procedure typically yields low amounts of highly purified fractions; for instance, two arabinogalactan-type polysaccharides (MCP-1a and MCP-2b) were obtained with yields of 0.51% and 0.35%, respectively [39]. For preliminary bioactivity screening, partially purified fractions obtained through alcohol precipitation and single-step chromatography may suffice. Throughout the purification process, mild conditions (moderate temperature and near-neutral pH) should be maintained to preserve glycosidic bond integrity and native polysaccharide conformation.

## Structural characteristics of maca polysaccharides

**Fig. 1:**
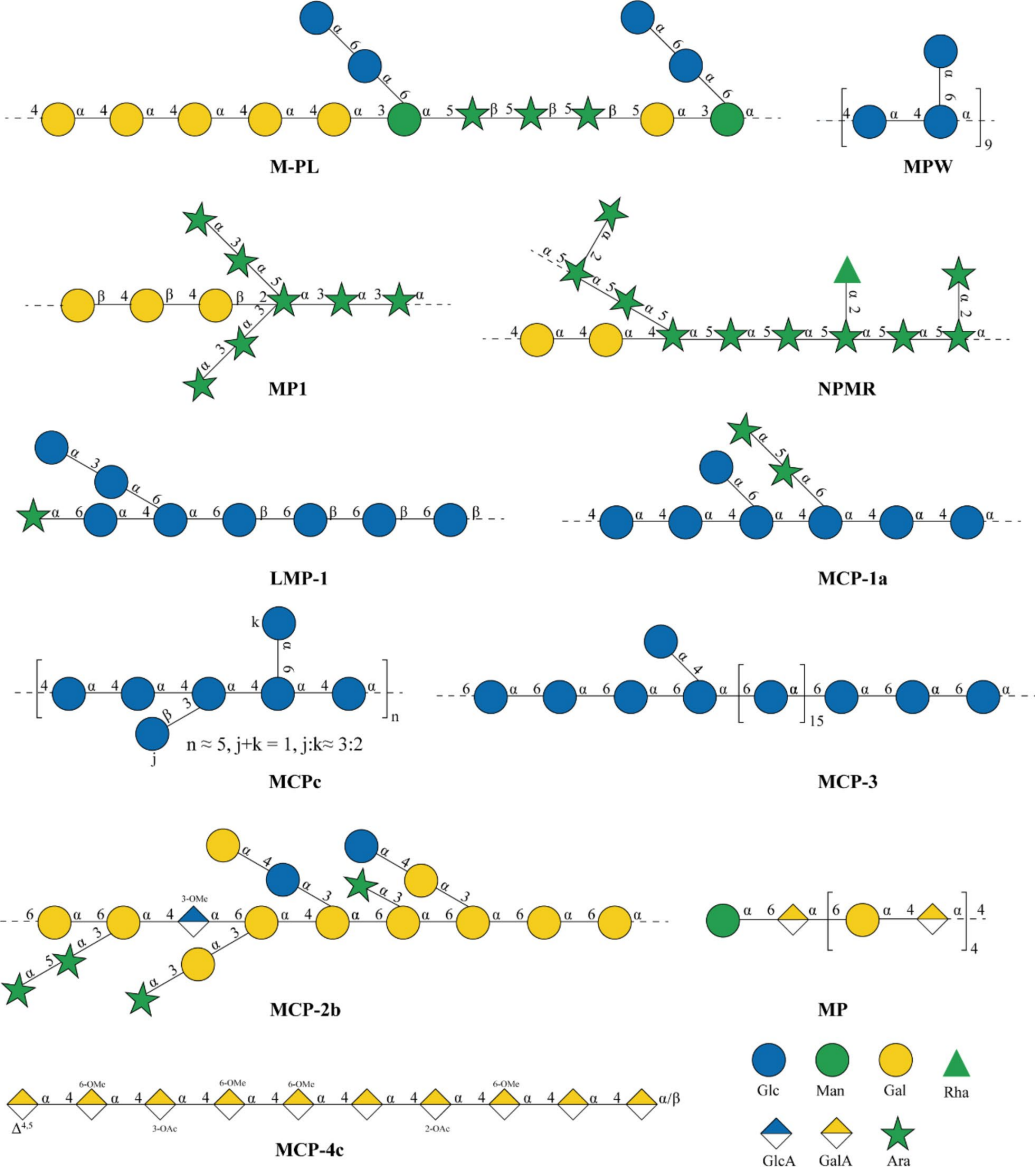
Schematic diagram of the structure of maca polysaccharide

Maca polysaccharides, as one of the major bioactive macromolecular fractions, exhibit significant structural heterogeneity and complexity (Fig. 1 and Table 1). Existing studies indicate that maca polysaccharides are not a single chemical entity but a complex mixture of various structural types, including α-glucans, pectin polysaccharides (homogalacturonans and rhamnogalacturonans), arabinogalactans, and mannose-containing heteropolysaccharides [38, 39]. This structural diversity arises from variations in glycosidic linkage patterns, branching degrees, monosaccharide composition ratios, and chemical modifications (such as methylation and acetylation), which collectively define the unique structural characteristics of maca polysaccharides.

**Table 1:** Structural characteristics of maca polysaccharides

Name	Extraction and purification	Structural type	Monosaccharide composition	Glycosidic bond type	Molecular weight (kDa)	References
MCPa-d	HWE; DEAE-52; Sephadex G-50, G-100	α-Glucan	Glc	(1 → 4)-α-D-Glcp, C-3 and C-6 branching	3.1–14.4	[43]
MCP-3	HWE; DEAE-52; Sephadex G-50, G-100	α-Glucan	Glc	1,6-α-D-Glcp, 1,3,6-α-D-Glcp, T-α-D-Glcp (O-3 branching)	1951.0	[38]
MCP-1a	HWE; DEAE-52; Sephadex G-50, G-100	Heteropolysaccharide	Glc:Ara (34.57: 1)	1,4-α-D-Glc,1,4,6-α-D-Glc, side chain: T-α-L-Ara,1,5-α-L-Ara, T-α-D-Glc (O-6 position)	6.6	[39]
LMP-1	HWE; DEAE-52; Sephadex G-100	Heteroglucan	Glc:Ara (7.03:1.08)	→ 4)-α-D-Glcp-(1 → , → 6)-α-D-Glcp-(1 → , → 3)-α-D-Glcp-(1 → , β-D-Araf-(1 → , O-6-branched	10.1	[44]
MC-1	HWE; DEAE-Sepharose; Sephadex G-50	Heteroglucan	Ara:Man:Glc:Gal (26.21:11.81:53.66:8.32)	→ 5)-α-L-Ara-(1 → , → 3)-α-L-Man-(1 → , → 2, 6)-α-L-Man-(1 → , α-D-Glc-(1 → , → 4)-α-D-Glc-(1 → , → 6)-α-D-Glc-(1 → , → 6)-β-D-Gal-(1 →	11.3	[45]
MC-2	HWE; DEAE-Sepharose; Sephadex G-50	Polysaccharide (triple helix conformation)	Ara:Man:Glc:Gal (20.9:4.5:71.9:2.7)	→ 5)-α-L-Ara-(1 → , → 3)-α-L-Man-(1 → , → 2, 6)-α-L-Man-(1 → , α-D-Glc-(1 → , → 4)-α-D-Glc-(1 → , → 6)-α-D-Glc-(1 → , → 6)-β-D-Gal-(1 →	9.8	
MPS-1	HWE; DEAE-52; Sephadex G-100	Mixed glucan (α/β-pyranose)	Xyl:Ara:Gal:Glc (1:1.7:3.3:30.5)	α-pyranose	7.6	[42]
MPS-2			Ara:Gal:Glc (1:1.3:36.8)	α-D-glucopyranose and β-D-glucopyranose	6.7	
MP1	HWE (100 °C); DEAE-52; Sephacryl S-500	Type I arabinogalactan	Ara:Gal (2:1)	α-L-Ara-(1 → , → 3)-α-L-Ara-(1 → , → 2)-α-L-Ara-(1 → , → 5)-α-L-Ara-(1 → , → 4)-α-D-Gal-(1 →	467.0	[47]
MCP-2b	HWE; DEAE-52; Sephadex G-50, G-100	Type II arabinogalactan (containing glucuronic acid)	Ara,Gal,GlcA	Skeleton: 1,6-β-D-Gal, 1,3,6-β-D-Gal, with 1,3-β-D-GlcpA-4-OMe insertion; Side chains: 1,3-β-D-Gal, T-β-D-Gal, T-α-L-Ara, 1,5-α-L-Ara, trace amounts of 1,4-β-D-Glc, T-β-D-Glc	49.4	[39]
MCP-4c	HWE; DEAE-52; Sephadex G-50, G-100	Acidic heteropolysaccharide	GalA	α/β-D-GalpA-(1 → , T-α-D-Δ^4,5^-GalpA-(1 → , → 4)-α-D-GalpA-(6-OMe) -(1 → , → 4)-α-D-GalpA-(3-OAc)-(1 → , → 4)-α-D-GalpA-(2-OAc) -(1 → , → 4)-α-D-GalpA-(1 →	6.5	[38]
MP	HWE; Sephacryl S-100HR	Acidic heteropolysaccharide	GalA:Glc:Ara:Man:Gal:Rha (35.07:29.98:16.98:13.01:4.21:0.75)	β-1,3-Galp(A), β-1,3-Glcp, α-1,3-Manp alternating (5:4:1)	793.5	[13]

HWE = Hot water extraction. Extraction conditions varied across studies (typically 80–100 °C, 2–4 h, solid-to-liquid ratio 1:20–1:40); see original references for specific parameters. Monosaccharide abbreviations—Ara: arabinose; Gal: galactose; Glc: glucose; Man: mannose; Rha: rhamnose; Xyl: xylose; GalA: galacturonic acid; GlcA: glucuronic acid. Glycosidic linkage notation: Numbers indicate linkage positions; “T-” indicates terminal (non-reducing) residues; “ → ” denotes bond direction; α/β specify anomeric configuration

### Glucan and heteroglycan structures

Maca glucans exhibit structural diversity ranging from linear chains to highly branched architectures. Molecular weights range from 3.1 kDa to 1951.0 kDa, and this nearly 600-fold variation suggests the coexistence of different degrees of polymerization and branching patterns. The four glucans (MCPa-MCPd) obtained through systematic isolation, despite having different molecular weights (3.1–14.4 kDa), share core structural features of the (1 → 4)-glucose main chain and C-3, C-6 branches, suggesting a conserved framework construction pattern for maca glucans [43]. This structural conservation, in contrast to the variability in molecular weight, indicates that branching frequency and side chain length regulate the molecular weight of glucans.

Structural analysis of LMP-1 (1.01 × 10^4^ Da)﻿ reveals the complexity of maca glucan [44]. Methylation and NMR analyses revealed that LMP-1 is mainly composed of → 4)-α-D-Glcp-(1 → , → 6)-α-D-Glcp-(1 → , → 3)-α-D-Glcp-(1 → , and β-D-Araf-(1 → residues, with branching at the O-6 position of → 4,6)-α-D-Glcp-(1 → . This diverse glycosidic linkage pattern confers chain flexibility and conformational variability to the polysaccharide. MCP-1a (6.6 kDa) has a backbone composed of 1,4-α-D-Glc and 1,4,6-α-D-Glc, with side chains including T-α-L-Ara, 1,5-α-L-Ara, and T-α-D-Glc attached at the O-6 positions [39]. This O-6 branching pattern is similar to that of LMP-1, further confirming that O-6 is the preferred branching site in maca α-glucans.

The structural features of the ultra-high molecular weight α-glucan MCP-3 (1951.0 kDa) are particularly noteworthy [39]. The main chain contains both 1,6-α-D-Glcp and 1,3,6-α-D-Glcp units, with the latter linked to T-α-D-Glcp side chains at the O-3 position, forming a highly branched tree-like structure [38]. Although MCP-1a and MCP-3 differ in molecular weight by approximately 300-fold, both exhibit branched structures—MCP-1a at the O-6 position and MCP-3 at the O-3 position—indicating that branching is a common structural feature of maca α-glucans, though at different sites. The key structural difference is that the 1,3,6-α-D-Glcp disubstituted units in the MCP-3 main chain increase branching density, which may contribute to its ultra-high molecular weight. This observation suggests that the molecular weight and spatial conformation of maca polysaccharides may be modulated by the proportion of disubstituted units in the main chain.

MC-1 (11.3 kDa) and MC-2 (9.8 kDa) represent another class of structurally complex heteropolysaccharides [15, 45]. MC-1 and MC-2 share similar molecular weights and glycosidic bond types, including (1 → 5)-α-L-Ara, (1 → 3)-α-L-Man, (1 → 4)-α-D-Glc, and (1 → 6)-α-D-Glc. However, their monosaccharide compositions differ significantly: compared to MC-1, MC-2 contains higher glucose (71.9% vs. 53.66%) but lower arabinose (20.9% vs. 26.21%) and mannose (4.5% vs. 11.81%). Notably, MC-2 appears to adopt a triple-helix conformation as suggested by Congo red assay, a characteristic structure of β-1,3-glucans [45]. If confirmed, the presence of the triple-helix conformation is of great biological significance because this rigid ordered structure facilitates recognition and binding by immune receptors such as Dectin-1 [46].

The high-purity polysaccharide MP (793.5 kDa, 99.2% purity) exhibits distinctive structural features [13]. The main chain of this acidic heteropolysaccharide is formed by the alternating connection of three monosaccharide units—β-1,3-Galp(A), β-1,3-Glcp, and α-1,3-Manp—in a molar ratio of 5:4:1, constituting a ‘ternary alternating’ structure. This alternating arrangement pattern is relatively uncommon in plant polysaccharides, and its biosynthetic mechanism likely involves the coordinated action of multiple glycosyltransferases. Galacturonic acid (35.07%) as the dominant component not only confers acidic characteristics and negative charge to the polysaccharide, but may also influence its binding to proteins and metal ions through electrostatic interactions. The structural features of MP, in contrast to pure α-glucan, reflect the diversity of maca polysaccharide synthesis pathways.

### Arabinogalactan

Arabinogalactans represent one of the structurally diverse types of maca polysaccharides, encompassing both type I and type II variants. MP1 (467 kDa) was characterized as an arabinogalactan from maca, with a 2:1 molar ratio of arabinose to galactose [47]. This arabinose-enriched composition suggests abundant arabinan side chains, which is a characteristic feature often observed in AG-I structures associated with pectin domains [48]. AG-I typically has a backbone of β-1,4-linked galactose, with arabinose side chains attached at the O-3 positions, and is often present as a side chain component of rhamnogalacturonan-I (RG-I) in pectin domains. The relatively high molecular weight of MP1 suggests either an extended main chain or a moderate degree of branching.

MCP-2b (49.4 kDa) represents a type II arabinogalactan (AG-II) isolated from maca [39, 49]. Its backbone consists of 1,6-β-D-Gal and 1,3,6-β-D-Gal units, representing a variant of the typical AG-II structure (which features a β-1,3-galactan main chain with β-1,6-galactan side chains). The side chains terminate with 1,3-β-D-Gal, T-β-D-Gal, and arabinose residues (T-α-L-Ara, 1,5-α-L-Ara), forming a highly branched tree-like structure. A distinctive structural feature is the insertion of 4-O-methylglucuronic acid (1,3-β-D-GlcpA-4-OMe) units within the backbone, a modification that is relatively uncommon in arabinogalactans. The introduction of glucuronic acid confers partially acidic character to the polysaccharide, and its methylation may influence interactions with cell wall proteins such as arabinogalactan proteins (AGPs). This structural feature raises the possibility that MCP-2b may originate from a proteoglycan complex, potentially as a glycan moiety covalently linked to proteins.

LMLP (58.4 kDa), isolated from maca leaves, exhibits tissue-specific structural features [50]^.^ The monosaccharide composition shows that arabinose and galactose together account for approximately 70%, with an Ara:Gal ratio of about 1:1.4. This intermediate ratio, combined with the presence of rhamnose, suggests a complex arabinogalactan structure that may share features of both AG-I and AG-II types. This intermediate composition suggests that LMLP may be either a mixture of type I and type II arabinogalactans, or a structural variant with intermediate characteristics. The presence of rhamnose (1.15%) suggests that LMLP may be associated with the hairy region side chains of pectin.

Compared with root polysaccharides [MP1 (Ara:Gal = 2:1) and MCP-2b (typical AG-II)] and leaf polysaccharides [LMLP (Ara:Gal ≈ 1:1.4)], maca arabinogalactans exhibit distinct tissue-specific structural distribution. This difference may stem from distinct physiological requirements of different tissues. Roots, as storage organs, may accumulate type I arabinogalactans; leaves, as photosynthetic organs, may require type II arabinogalactans and their protein complexes to support cell wall mechanical strength and signal transduction. → 5)-α-L-Ara-(1 → linkages were detected in all arabinogalactans examined, representing a characteristic structural motif of arabinan side chains that extend from the galactan backbone.

### Pectin structure

Pectin, an important structural component of plant cell walls, represents a significant fraction of maca polysaccharides. Five homogalacturonans (MCP-4a to MCP-5b, 6.5–36.1 kDa), first isolated from Peruvian yellow maca, represent the typical pectin ‘smooth region’ structure [38]. These polysaccharides form a linear backbone of α-1,4-linked galacturonic acid residues, with partial methyl esterification (GalpAOMe), characteristic of low-methoxyl pectin. Fine structural analysis of MCP-4c reveals the complexity of maca pectin modifications: the backbone contains 6-O-methyl esterification and acetylation at C-2 and C-3 positions (2-OAc, 3-OAc), along with terminal unsaturated galacturonic acid residues (T-α-D-Δ^4,5^-GalpA). These multi-site chemical modifications modulate the physicochemical properties of pectin; methylation reduces carboxyl charge density, while acetylation influences interchain hydrogen bonding, collectively affecting gel properties and solubility [51].

The presence of rhamnose and galacturonic acid in multiple maca polysaccharide fractions suggests the occurrence of rhamnogalacturonan (RG) domains. MP21 (3.68 × 10^5^ Da) is mainly composed of rhamnose, arabinose, and galactose in a molar ratio of 1:4.84:5.34, and the high arabinose and galactose content is consistent with the hairy region side chain composition of RG-I pectin [41]. RG-I is characterized by a backbone of repeating [→ 2)-α-L-Rhap-(1 → 4)-α-D-GalpA-(1 →] disaccharide units, with rhamnose residues bearing highly branched arabinan and galactan side chains at the O-4 position. Although detailed glycosidic linkage analysis of MP21 is lacking, its monosaccharide composition and high molecular weight suggest an RG-I type pectin with abundant side chains. Conformational analysis revealed that MP21 adopts an intermediate conformation between spherical and random coil, potentially reflecting a balance between the rigid backbone and flexible side chains.

Compared with the linear homomorphic galacturonic acid glycans (MCP-4 series) and possibly highly branched rhamnol galacturonic acid glycans (MP21), maca pectin shows a continuous distribution from the smooth zone to the hair zone in terms of structural organization. This structural diversity reflects the spatial heterogeneity of pectin within the cell wall and suggests functional differentiation: smooth regions contribute to intercellular adhesion, while hairy regions may participate in signaling and cross-linking with cellulose and hemicellulose.

### Mannan domains

Although mannose content is relatively low in maca polysaccharides (4.5–13.01%), the diversity of its structural roles indicates its functional significance [13, 15, 45]. Mannose participates in maca polysaccharide construction in at least three forms. First, it serves as a side chain branching point; for example, (1 → 2,6)-α-L-Man in MC-1 functions as a disubstituted residue connecting two side chains [15]. Second, (1 → 3)-α-L-Man residues may form short oligosaccharide extensions. Third, in MP, α-1,3-mannose alternates with β-1,3-galacturonic acid and β-1,3-glucose as a backbone building unit [13]. This diversity suggests that mannose, despite its low abundance, may function as a structural hub contributing to the three-dimensional organization of these polysaccharides [52].

Notably, although (1 → 3)-α-L-Man and (1 → 2,6)-α-L-Man linkages have been detected in multiple studies, pure mannan or glucomannan has not been isolated from maca to date [7]. Mannose consistently occurs as a component of heteropolysaccharides, consistent with the polysaccharide composition pattern of the Brassicaceae family to which maca belongs. Brassicaceae cell wall polysaccharides are typically characterized by complex heteropolysaccharides, with mannose residues incorporated into glucomannan rather than existing as pure mannan or galactomannan storage polysaccharides common in legume seeds [53, 54]. This phylogenetic conservation suggests that maca polysaccharide structure may be constrained by the glycosyltransferase gene repertoire encoded in the Brassicaceae genome [55].

The considerable range of mannose content observed among isolated fractions, from 4.5% in MC-2 to 13.01% in MP, underscores both the methodological influence of extraction protocols and the intrinsic structural diversity inherent to maca polysaccharides. This heterogeneity may reflect regional differences in mannose distribution within cell wall domains. Additionally, selective extraction of specific polysaccharide fractions and variations in growth conditions such as origin, altitude, and climate could influence polysaccharide biosynthesis. From a structure–function perspective, mannose residues can be recognized by the mannose receptor CD206 expressed on macrophages and dendritic cells. Therefore, despite their relatively low abundance (4.5–13%), mannose residues contribute to the immunomodulatory activity of maca polysaccharides, as demonstrated by receptor blocking experiments [15].

### Structural regularities and influencing factors

Based on the structural data summarized above, several regularities and influencing factors can be identified for maca polysaccharides.

Relationship between molecular weight and structural type. Analysis of the characterized maca polysaccharides reveals a general correlation between molecular weight distribution and structural type. Low-molecular-weight fractions (generally < 20 kDa) are predominantly α-glucans sharing a conserved backbone of (1 → 4)-α-D-Glcp with branches at C-3 and C-6, as exemplified by MCPa–MCPd (3.1–14.4 kDa) [43] and MCP-1a (6.6 kDa) [39]. Medium-molecular-weight fractions (approximately 40–500 kDa) exhibit greater structural diversity, encompassing type II arabinogalactans such as MCP-2b (49.4 kDa), type I arabinogalactans such as MP1 (467 kDa), and RG-I type pectins such as MP21 (368 kDa) [39, 41, 47]. Ultra-high-molecular-weight fractions (> 1000 kDa), represented by MCP-3 (1951 kDa), are characterized by highly branched α-glucan architectures with increased proportions of disubstituted residues (e.g., 1,3,6-α-D-Glcp) that contribute to their extended chain length [38]. It should be noted that some structural types, such as homogalacturonans (6.5–36.1 kDa), span across molecular weight categories, indicating that molecular weight alone does not fully determine structural type.

Influence of extraction methods on structural characteristics. A comparative study examining four extraction methods for maca polysaccharides demonstrated that the extraction conditions significantly affect the physicochemical properties of the resulting polysaccharides [34]. Hot water extraction at elevated temperatures (90 °C) promotes the decomposition of high-molecular-weight polysaccharides into low-molecular-weight oligosaccharides and monosaccharides, resulting in higher water solubility but potentially altered structural integrity. In contrast, enzyme-assisted extraction yields polysaccharides with lower protein contamination (1.71% vs. 7.71% for ultrasound-assisted extraction), although with reduced overall recovery. Ultrasound-assisted extraction under milder conditions (50 °C) has been shown to preserve structural integrity while achieving higher yields [24]. Furthermore, the fractionation strategy determines which structural types are isolated: neutral α-glucans elute unretained during anion-exchange chromatography, whereas acidic pectins and arabinogalactans containing uronic acids are selectively retained [39, 41].

Predominant structural type and main chain characteristics. Based on the frequency of isolation and characterization reported in the literature (Table 1), α-glucans have been most frequently isolated and characterized from maca, suggesting they may represent a major structural type. The conserved α-glucan backbone consists of (1 → 4)-α-D-Glcp residues, with branching occurring primarily at the O-6 position through (1 → 4,6)-α-D-Glcp units, and less frequently at the O-3 position via (1 → 3,6)-α-D-Glcp units [38, 39, 43, 44]. This backbone architecture, combined with variable branching frequency and side chain composition (including terminal arabinose and glucose residues), accounts for the wide molecular weight range observed among maca α-glucans (3.1–1951 kDa). Pectic polysaccharides, including homogalacturonans and rhamnogalacturonans, have also been frequently characterized as the second prevalent structural type [38, 41].

## Biological activity of maca polysaccharides

Maca polysaccharides exhibit a broad spectrum of pharmacological activities, including immunomodulation, antioxidant effects, anti-fatigue properties, and hepatoprotection. Existing studies are mainly based on in vitro cell models and animal experiments. To provide a clearer mechanistic framework, this section reorganizes the biological activities according to three criteria: (1) research depth and mechanistic clarity, (2) frequency of published studies, and (3) pathway interconnections. Immunomodulatory activity is presented first due to its relatively well-characterized receptor-signaling mechanisms. Antioxidant activity and hepatoprotection are discussed together because they share the Nrf2/Keap1-HO-1 pathway as the central mechanism. Anti-fatigue activity follows, with emerging activities presented last. Table 2 summarizes the key signaling pathways, biomarkers, and representative polysaccharide fractions associated with each biological activity.

**Table 2:** Summary of biological activities, signaling pathways, and key biomarkers of maca polysaccharides

Biological activity	Core signaling pathways	Key biomarkers	Representative fractions	References
Immunomodulation	TLR2/TLR4-NF-κB; MAPK	NO, TNF-α, IL-6, IL-1β; CD4⁺/CD8⁺ ratio	MC-1, MC-2, LMP-1, MCP2, MP21	[15, 39, 41, 44, 45, 56–60]
Antioxidant and Hepatoprotection	Nrf2/Keap1-HO-1; AhR/STAT3	SOD, GSH-Px, CAT, MDA; AST, ALT	MP-1, MP	[61–63]
Anti-fatigue	Energy metabolism	Liver/muscle glycogen, blood lactate, blood urea nitrogen (BUN), LDH	MP, MPS-1, MPS-2, MCP	[13, 17, 42]
Gut health and Anti-inflammation	TLR4; Gut microbiota	SCFAs, ZO-1, occludin; TNF-α, IL-10	MCP-3, MC-1, MC-2	[38, 64]

### Immune regulatory activity

**Fig. 2:**
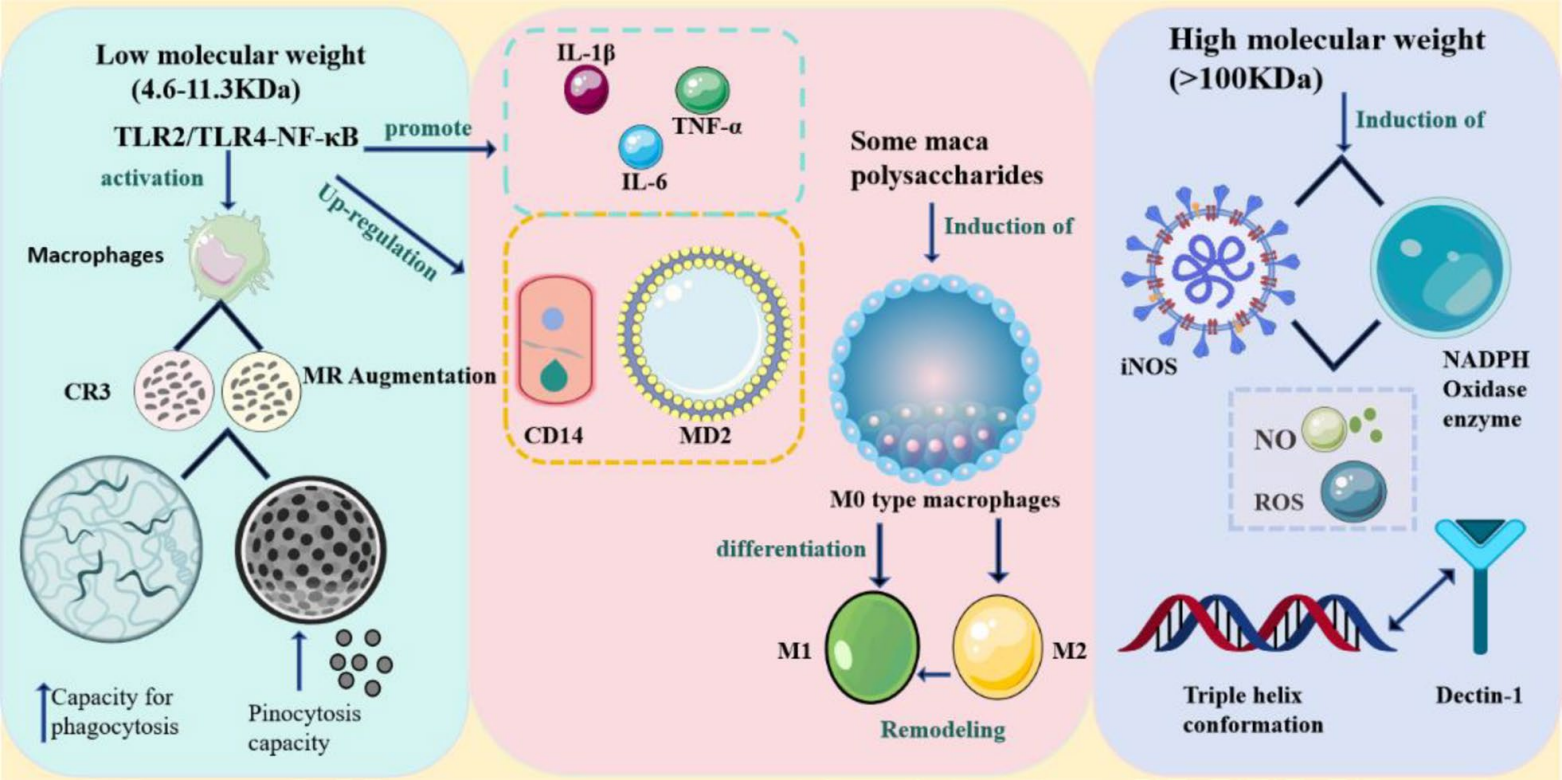
Immunomodulatory mechanism of maca polysaccharides

Maca polysaccharides activate immune cells through pattern recognition receptors (PRRs), including Toll-like receptors 2 and 4 (TLR2, TLR4), complement receptor 3 (CR3), and mannose receptor (CD206). These receptors converge on downstream nuclear factor kappa B (NF-κB) and mitogen-activated protein kinase (MAPK) signaling cascades, leading to cytokine production and immune cell activation (Fig. 2). The immunomodulatory effects can be classified into innate immunity activation and adaptive immunity regulation.

#### Innate immunity: Macrophage activation

Low-molecular-weight fractions (9.8–11.3 kDa) activate macrophages predominantly through the TLR2/TLR4-NF-κB signaling pathway. LMP-1 (10.1 kDa) upregulates CD14 and MD2 expression, enhancing TLR signaling [44]. MC-1 (11.3 kDa) activates macrophages and enhances cytokine secretion, with specific receptor-mediated mechanisms discussed in Sect. 5.2 [15]. The activated macrophages exhibit increased secretion of pro-inflammatory cytokines (interleukin-1 beta [IL-1β], TNF-α, IL-6) and enhanced phagocytic capacity [44, 45].

High-molecular-weight fractions show distinct activation patterns. MP21 (368 kDa), which adopts an intermediate conformation between spherical and random coil, predominantly induces inducible nitric oxide synthase (iNOS) expression and promotes NO and reactive oxygen species (ROS) production [41, 65]. Spatial conformation influences immune recognition. MC-2 (9.8 kDa) exhibits potent macrophage-activating activity, inducing M0-type macrophages to differentiate into the M1 phenotype and reprogramming M2-type macrophages toward M1 polarization [45]. The structural basis for this enhanced activity is analyzed in Sect. 5.4.

#### Adaptive immunity: T cell regulation

In cyclophosphamide-induced immunosuppression models, maca polysaccharide administration restored immune organ indices, increased white blood cell counts, and enhanced lymphocyte proliferation with an increased proportion of cells entering the S phase and G2/M phase [56]. The cluster of differentiation 4 positive (CD4⁺) T cell percentage and CD4⁺/CD8⁺ ratio were significantly elevated, accompanied by increased serum levels of T helper type 1 (Th1) cytokines (interferon-gamma [IFN-γ], TNF-α, interleukin-2 [IL-2]) and decreased T helper type 2 (Th2) cytokine interleukin-4 (IL-4). T-bet expression was upregulated while GATA-3 was downregulated, demonstrating that maca polysaccharides promote a Th1-biased immune response [57]. It should be noted that these studies used severe immunosuppression models, and the immunomodulatory effects under normal physiological conditions require further evaluation [16].

Molecular weight influences adaptive immunity regulation. Among four fractions (MCP1–MCP4) obtained by graded alcohol precipitation, the medium-molecular-weight fraction MCP2 (219–338 kDa) exhibited the strongest activity in promoting CD4⁺ T cell proliferation and IFN-γ secretion, whereas the low-molecular-weight fraction MCP4 (12–16 kDa) showed significantly weaker activity [58].

#### Tumor immunomodulation

MCP2 combined with 5-fluorouracil (5-FU) in Lewis lung cancer models partially restored 5-FU-induced CD4⁺ T cell proliferation inhibition and delayed tumor growth [58, 59]. Furthermore, maca polysaccharides and their cationic derivatives can reprogram tumor-associated macrophages (TAMs) from the M2 to M1 phenotype, increasing pro-inflammatory cytokine levels (IL-12, TNF-α) and IFN-γ in the tumor microenvironment [60]. These findings provide preliminary experimental evidence from preclinical models**.** It should be noted that no clinical trials specifically evaluating maca polysaccharides in cancer adjuvant therapy have been reported to date. Future clinical translation would require addressing bioavailability, targeted delivery, and safety profile challenges through well-designed human studies.

### Antioxidant activity and hepatoprotection

**Fig. 3:**
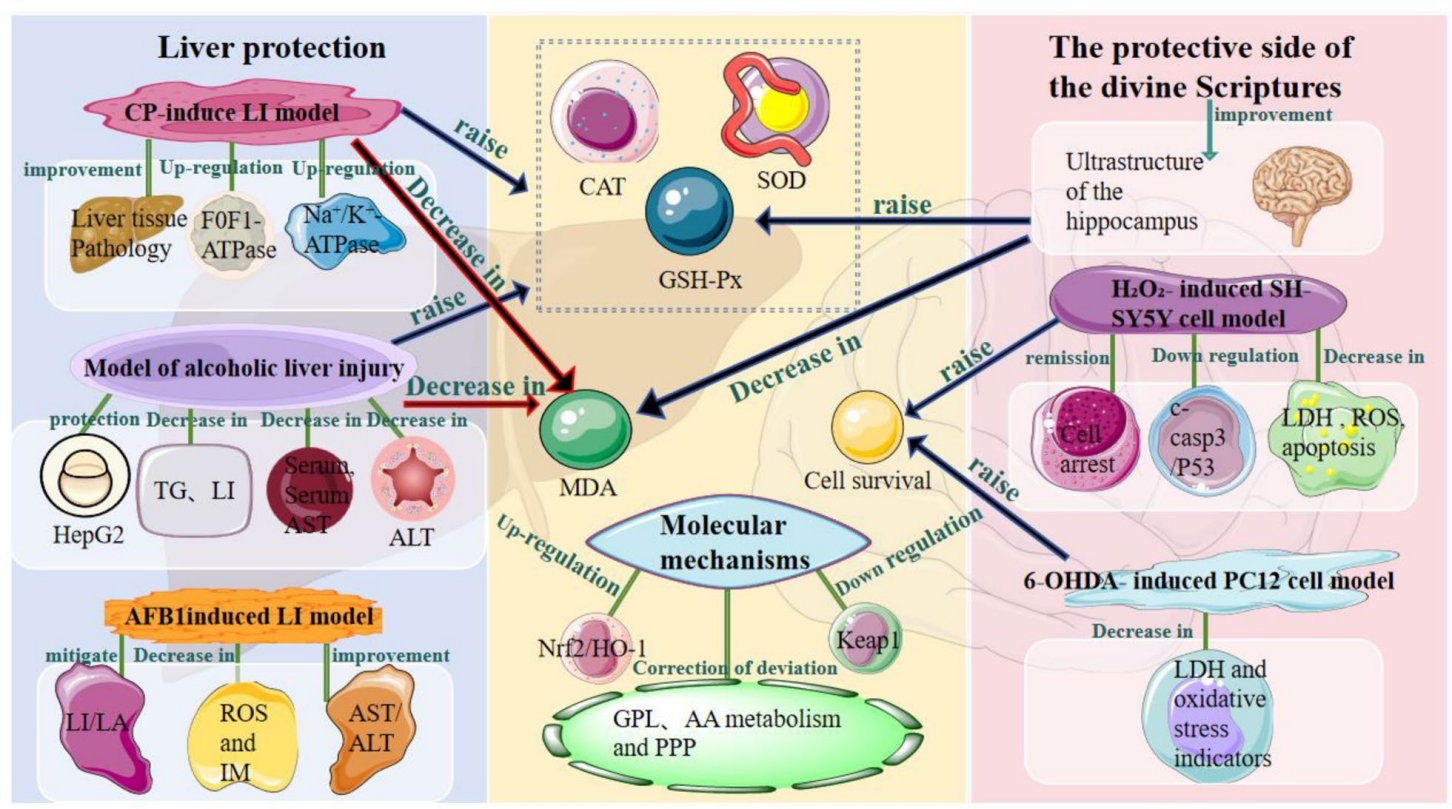
Mechanism of antioxidant and hepatoprotective effects of maca polysaccharides

Antioxidant activity and hepatoprotection share the nuclear factor erythroid 2-related factor 2 (Nrf2)/Kelch-like ECH-associated protein 1 (Keap1)-heme oxygenase-1 (HO-1) pathway as their central mechanism (Fig. 3). Under oxidative stress conditions, maca polysaccharides activate Nrf2 signaling, leading to upregulation of antioxidant enzymes. This mechanistic overlap justifies the combined discussion of these two activities.

#### Antioxidant mechanisms

In vitro, heteropolysaccharides containing diverse monosaccharides (Rib, Rha, Ara, Xyl, Man, Glc, and Gal) exhibit stronger scavenging capacities for hydroxyl radicals, superoxide anions, and DPPH radicals compared to homogeneous glucans composed solely of glucose [47]. However, in vitro antioxidant results do not directly translate to in vivo effects, as they cannot adequately reflect absorption, metabolism, and interactions with the endogenous antioxidant system.

In vivo, maca polysaccharides primarily function by enhancing endogenous antioxidant defense. In cyclophosphamide-induced hepatotoxicity models, maca polysaccharide administration increased hepatic superoxide dismutase (SOD), glutathione peroxidase (GSH-Px), and catalase (CAT) activities while reducing malondialdehyde (MDA) levels, consistent with improved liver histopathology. Mechanistically, Western blot and transcriptional assays revealed upregulation of Nrf2 and HO-1 with concurrent downregulation of Keap1 [61]. Metabolomic analysis further demonstrated corrections in glycerophospholipid metabolism, arachidonic acid metabolism, and the pentose phosphate pathway.

#### Hepatoprotective effects

The hepatoprotective effects of maca polysaccharides have been validated in multiple liver injury models: In alcohol-induced liver injury models, MP-1 (1067.3 kDa) protected HepG2 cells from ethanol-induced damage, reduced serum and hepatic triglyceride levels, decreased aspartate aminotransferase (AST) and alanine aminotransferase (ALT), enhanced hepatic SOD, GSH-Px, and glutathione S-transferase (GST) activities, and alleviated liver inflammation and steatosis [62]. In cyclophosphamide-induced hepatotoxicity models, maca polysaccharides reduced serum transaminases, improved liver histopathology, enhanced antioxidant enzyme activities (SOD, GSH-Px, CAT), and upregulated energy metabolism-related enzymes including F0F1-ATPase, Na⁺-K⁺-ATPase, and Ca^2^⁺-Mg^2^⁺-ATPase [61]. In aflatoxin B1 (AFB1)-induced hepatotoxicity models, maca polysaccharides (0.4–1.6 g/kg BW/d, 28 days) alleviated liver tissue damage and lipid accumulation, reduced oxidative stress and inflammatory markers, and improved serum transaminase levels through modulation of both the Nrf2/Keap1 and aryl hydrocarbon receptor (AhR)/signal transducer and activator of transcription 3 (STAT3) signaling pathways [63].

The common mechanistic feature across these models is activation of the Nrf2/Keap1-HO-1 pathway combined with improvement of energy metabolism. Existing research has mainly focused on acute or subacute injury models, and the long-term effects on chronic liver disease remain to be studied.

#### Neuroprotective effects

Maca polysaccharides also demonstrate neuroprotective potential through antioxidant mechanisms. In D-galactose-induced aging mouse models, maca polysaccharides improved hippocampal ultrastructure, increased brain GSH-Px activity, and reduced MDA levels. In H₂O₂-induced SH-SY5Y cell models, maca polysaccharides increased cell survival, reduced lactate dehydrogenase (LDH) leakage and ROS levels, inhibited apoptosis, and downregulated cleaved caspase-3 and p53 expression [66]. In 6-OHDA-induced PC12 cell models, maca leaf methanol extract increased cell viability (by 31–60%) and reduced LDH leakage (by 31–48%) [7, 67]. In addition, maca powder administration improved cognitive function in middle-aged mice, associated with enhanced mitochondrial respiratory function and upregulation of autophagy-related proteins (LC3, Atg7, Beclin-1) [68]. However, the specific contribution of polysaccharide components to these neuroprotective effects requires further investigation using purified fractions.

### Anti-fatigue activity and energy metabolism regulation

**Fig. 4:**
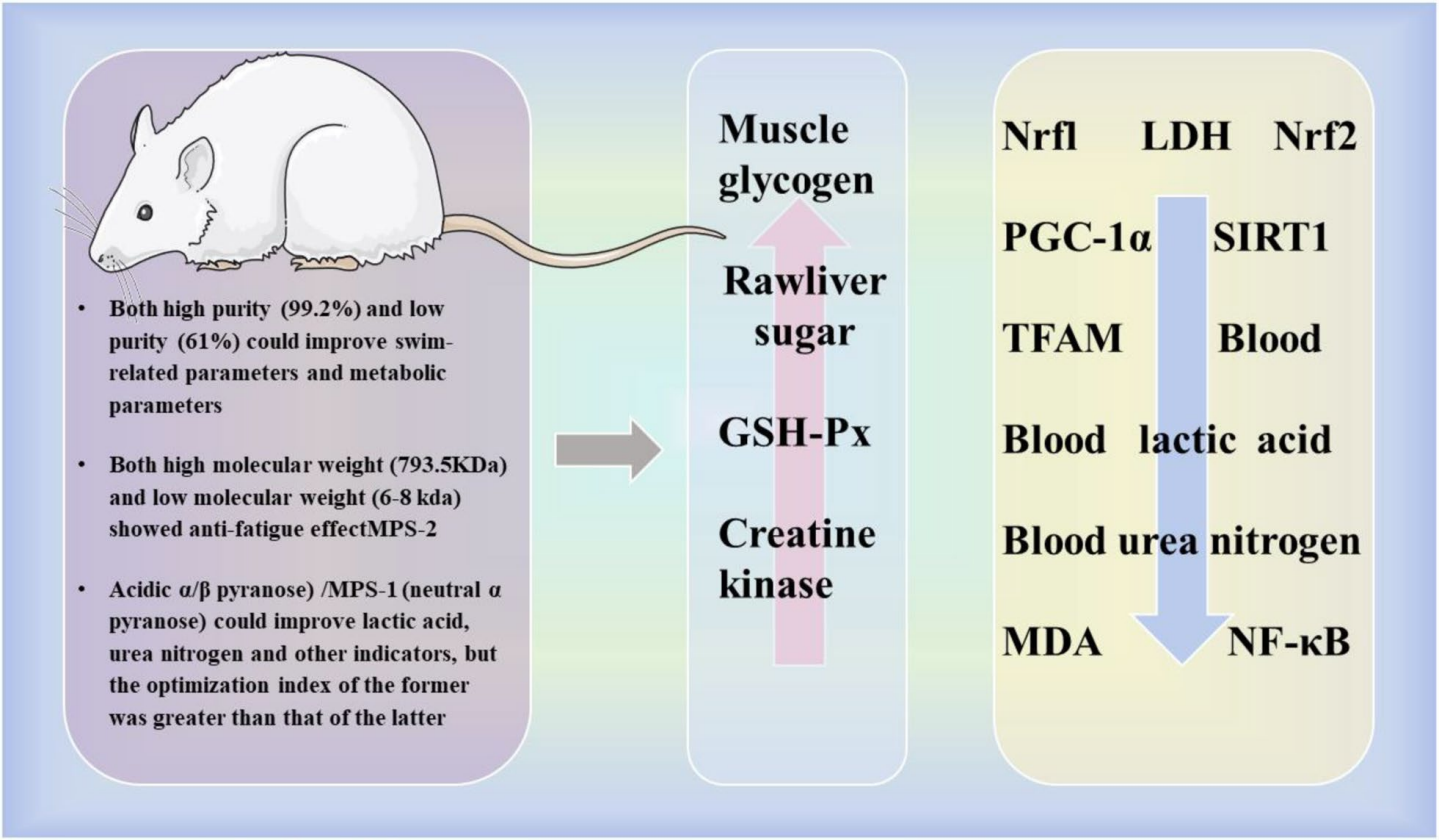
Mechanism of anti-fatigue action of maca polysaccharides

Anti-fatigue activity represents one of the traditional applications of maca. The underlying mechanisms involve enhancement of energy substrate storage, acceleration of metabolic waste clearance, and reduction of oxidative stress (Fig. 4).

#### Anti-fatigue effects from exercise models

Maca polysaccharides exhibit anti-fatigue activity across different molecular weight ranges and purity levels. High-purity acidic polysaccharide MP (793.5 kDa, 99.2% purity) at doses ≥ 50 mg/kg BW/d for 30 days prolonged weight-loaded swimming time, increased average swimming speed within the first 5 min, and improved serum biochemical parameters in mice [13]. Low-molecular-weight fractions MPS-1 (7.6 kDa) and MPS-2 (6.7 kDa) also exhibited anti-fatigue effects. MPS-2, containing both α- and β-pyranose configurations, demonstrated superior improvements in blood lactate, urea nitrogen, LDH activity, and liver glycogen levels compared to MPS-1, which contains only α-pyranose [42]. Furthermore, crude polysaccharide extract MCP (purity ~ 61%, protein content 4.46%) also improved swimming parameters and metabolic indicators, suggesting that anti-fatigue activity is not entirely dependent on high purity levels [17].

#### Metabolic mechanisms

Multiple studies consistently reported increased hepatic glycogen content, decreased blood lactate and blood urea nitrogen levels, enhanced GSH-Px activity, and reduced MDA levels following maca polysaccharide administration [13, 17, 42]. These changes indicate that maca polysaccharides support energy supply, accelerate metabolite clearance, and reduce oxidative stress during exercise.

At the molecular level, one study used maca powder (rather than purified polysaccharides) to supplement rats at 40 mg/kg BW/d for 21 days [69]. Results demonstrated prolonged swimming time to exhaustion, decreased serum lactate and tissue MDA levels, and increased muscle GSH-Px activity. Concurrently, skeletal muscle NF-κB expression was downregulated, while mitochondrial biogenesis-related proteins (nuclear respiratory factor 1 [Nrf1], Nrf2, peroxisome proliferator-activated receptor gamma coactivator 1-alpha [PGC-1α], sirtuin 1 [SIRT1], and mitochondrial transcription factor A [TFAM]) were upregulated. However, given the material differences between whole maca powder and purified polysaccharide isolates, direct attribution of these molecular mechanisms to polysaccharide components requires further validation using isolated fractions.

### Other biological activities

#### Gut microbiota modulation and intestinal barrier protection

Maca polysaccharides exhibit prebiotic potential and intestinal barrier protective effects based on in vitro evidence. In vitro fermentation experiments demonstrated that both α-glucan (MCP-3) and homogalacturonan fractions could be fermented by human gut microbiota to produce short-chain fatty acids (SCFAs), accompanied by increased abundance of beneficial Bacteroidetes [38]. In Caco-2 intestinal epithelial cell models, MC-1 and MC-2 inhibited LPS-induced intestinal barrier damage by upregulating tight junction proteins (zonula occludens-1 [ZO-1] and occludin) and regulating TLR4 expression, while suppressing pro-inflammatory cytokines (TNF-α, IL-8, IFN-γ) and increasing anti-inflammatory IL-10 secretion [64]. These findings demonstrate that maca polysaccharides contribute to intestinal health in vitro, although in vivo validation remains limited.

#### Context-dependent anti-inflammatory effects

The anti-inflammatory activity of maca polysaccharides is closely associated with their immunomodulatory properties and exhibits context-dependent characteristics. Maca polysaccharides regulate the balance between pro-inflammatory and anti-inflammatory cytokines, primarily through the NF-κB signaling pathway [41, 44], with STAT3 involvement observed in hepatoprotective contexts [63]. Under immunosuppression or tissue damage conditions, maca polysaccharides promote Th1/M1 polarization, while exhibiting anti-inflammatory effects in LPS-induced inflammation models [45, 56]. This bidirectional regulatory capacity may reflect the involvement of different polysaccharide structural types engaging distinct receptor-signaling pathway combinations.

#### Potential hypoglycemic activity

Preliminary studies suggest that maca polysaccharides possess hypoglycemic potential through α-glucosidase inhibitory activity. Four glucans (MCPa–MCPd) with molecular weights ranging from 3.1 to 14.4 kDa demonstrated inhibitory effects on α-glucosidase activity in vitro [43]. However, direct in vivo antidiabetic effects require further validation.

## Analysis of the structure–activity relationship of maca polysaccharides

The biological activity of polysaccharides is not a simple reflection of their chemical structure, but emerges from the integrated effects of structural parameters on receptor recognition, signal transduction, and downstream biological processes. Maca polysaccharides exhibit significant structural heterogeneity, with molecular weights spanning from approximately 3 kDa to 1951 kDa, predominantly α-glycosidic linkages (though β-linkages also occur), and diverse monosaccharide compositions. Understanding how these structural characteristics influence biological activities such as immunomodulation, anti-fatigue effects, and antioxidant capacity requires systematic analysis from a structure–function perspective.

### Molecular weight-dependent modulation of biological activity

Molecular weight is a key parameter regulating biological activity through its effects on solubility, diffusion, receptor-binding affinity, and conformational stability. In general, immunomodulatory activity exhibits a moderate molecular weight optimum pattern, with the range of approximately 10–1000 kDa often cited as favorable, although optimal values vary depending on polysaccharide type and assay system [70, 71]. This phenomenon can be explained by polyvalent receptor clustering and delivery efficiency. Low-molecular-weight polysaccharides may fail to achieve effective multivalent coordination and receptor aggregation. Conversely, high-molecular-weight species often exhibit reduced efficacy due to limited solubility, impaired diffusion, and decreased target cell accessibility [72]. Nevertheless, some low-molecular-weight fractions can still elicit immune responses when possessing favorable conformational features, charge density, or branching patterns [73]. This indicates that the molecular weight threshold is not absolute, but rather interacts with conformational and multivalent effects.

Graded fractionation studies of maca polysaccharides support this pattern. Water-extracted polysaccharides were separated into MCP1-MCP4 fractions (approximately 12–896 kDa). The medium-molecular-weight fraction MCP2 (219–338 kDa) exhibited the strongest activity in promoting CD4⁺ T cell proliferation and IFN-γ secretion, whereas MCP4 (12–16 kDa) showed significantly weaker activity [58]. This pattern is corroborated by studies on other plant polysaccharides. Astragalus polysaccharides were fractionated into APS-I (> 2,000 kDa), APS-II (~ 10 kDa), and APS-III (~ 300 Da) [73]. Both in vitro and in vivo screening demonstrated that APS-II exhibited the highest immunomodulatory activity, supporting the generality of the ‘medium Mw optimum’ principle.

Receptor contact mode may directly link molecular weight to activity. Low-molecular-weight fragments can more readily penetrate the extracellular matrix and access cell surface receptors or endocytic pathways. In contrast, high-molecular-weight and highly branched polysaccharides tend to amplify downstream signals through multivalent receptor clustering [74]. Notably, the low-molecular-weight fractions MC-1 (~ 11.3 kDa) and MC-2 (~ 9.8 kDa) exhibit potent macrophage-activating activity despite their relatively small size [15, 45]. This observation demonstrates that molecular weight alone does not determine immunomodulatory potential; other structural features including monosaccharide composition (Sect. 5.2) and spatial conformation (Sect. 5.4) also play critical roles in receptor recognition. The high-molecular-weight fraction MP21 (368 kDa) adopts an intermediate conformation between spherical and random coil. This architecture enables engagement with multiple receptor binding sites [41].

Notably, anti-fatigue activity appears less dependent on molecular weight than immunomodulatory activity. Both high-molecular-weight (MP, ~ 793.5 kDa) and low-molecular-weight fractions (MPS-1/MPS-2, ~ 6.7–7.6 kDa) demonstrated anti-fatigue effects in animal models [13, 42]. This suggests that anti-fatigue activity arises primarily from systemic modulation of energy metabolism and antioxidant pathways, rather than specific receptor-ligand interactions. Related studies have observed upregulation of mitochondrial biogenesis factors (PGC-1α, Nrf1, TFAM) and antioxidant pathway components (Nrf2/HO-1). These changes are consistent with enhanced glycogen reserves and improved lactate clearance. However, these studies primarily used whole maca powder or crude extracts rather than purified polysaccharides. Thus, extrapolation of these mechanisms to polysaccharide fractions requires caution [69].

### Effects of monosaccharide composition on receptor recognition

Monosaccharide composition influences the recognition specificity and binding affinity of polysaccharides to immune receptors. Specific monosaccharides exhibit preferential receptor recognition [75–77]. Mannose is recognized by the mannose receptor (MR/CD206), β-1,3/(1,6)-glucans by Dectin-1, and galactose-rich structures by galectins such as galectin-3. Pectin-type polysaccharides containing galactose or β-galactoside residues may also engage galectin-3 through their hairy region side chains. Glucuronic acid residues, being negatively charged, can modulate binding efficiency by altering chain charge density, hydration, and extensibility. Importantly, high glucose content does not guarantee Dectin-1 recognition. This receptor specifically requires β-(1 → 3)/(1 → 6) linkage patterns and appropriate higher-order conformations, particularly triple-helical structures [46].

In maca polysaccharides, mannose content is relatively low (approximately 4.5–13%). Nevertheless, its role in receptor recognition should not be overlooked. The immunostimulatory activity of MC-1 is partially mediated by the mannose receptor (CD206), with TLR2 and CR3 also implicated in signal transduction [15]. In studies on ginseng polysaccharides and others, mannose residues are involved in innate immune responses and pathogen uptake by binding to MR, which is consistent with the above observations [78, 79]. Notably, MC-1 contains → 2,6)-α-L-Man-(1 → residues, which serve as disubstituted branching points. This architecture may orient multiple recognition groups to form multivalent ligand clusters, potentially enhancing cooperative receptor binding.

The comparison between MC-1 and MC-2 provides direct evidence that monosaccharide composition influences biological activity [15, 45]. Both polysaccharides have similar molecular weights (11.3 vs. 9.8 kDa) and glycosidic bond types. However, MC-2 contains a higher proportion of glucose (71.9% vs. 53.66%) and lower proportions of arabinose (20.9% vs. 26.21%) and mannose (4.5% vs. 11.81%). Receptor blocking experiments demonstrated that MC-1 activates macrophages through TLR2, CR3, and the mannose receptor (CD206), with its higher mannose content (11.81%) potentially contributing to CD206-mediated recognition. In contrast, MC-2 exhibits stronger M1 polarization-inducing activity (Sect. 4.1.1), which correlates with its distinct monosaccharide profile. The putative triple-helix conformation of MC-2, as suggested by Congo red assay, may further enhance its immunomodulatory potency through improved receptor engagement (Sect. 5.4). Thus, compositional differences and spatial conformations are often intertwined, making it difficult to isolate their individual contributions to biological activity. Similar observations have been made with citrus polysaccharides CAVAP-I and CAVAP-II: both contain arabinose, mannose, glucose, and galactose, but differences in their proportions correlate with different immune-enhancing potentials [72].

The functional mechanisms of glucuronic acid are more diverse and merit separate consideration. As an acidic sugar, glucuronic acid imparts negative charge to the polysaccharide, alters solubility and chain extension, and thereby may increase effective receptor contact [80]. In pectin polysaccharide research, studies suggest a correlation between uronic acid content and immunomodulatory activity. When the proportion of uronic acid is approximately 20%–50%, optimal activity is generally observed [72]. This pattern is consistent with observations in maca polysaccharides. The high-purity acidic polysaccharide MP (GalA ≈35.07%) exhibits notable anti-fatigue activity [13]. Additionally, among low-molecular-weight fractions, MPS-2 containing both α- and β-pyranose configurations outperforms MPS-1 with only α-pyranose in anti-fatigue assays, suggesting that structural diversity may contribute to bioactivity [42]. Studies on pumpkin (*Cucurbita moschata*) have similarly demonstrated that pectic polysaccharides exhibit immunomodulatory activity. CMDP-4b, a pectic polysaccharide fraction, activates macrophages via TLR4 and CR3 receptors, triggering NF-κB and MAPK signaling pathways [81]. This enhanced activity is attributed to the combined effects of molecular weight, branching patterns, and uronic acid content.

### Glycosidic bond types and receptor recognition pathways

Glycosidic bond types shape higher-order conformations by influencing the rigidity, branching geometry, and charge distribution of the chain. β-(1 → 3)/(1 → 6)-glucans are among the most extensively studied immunomodulatory polysaccharides, particularly in the context of targeted drug delivery and tumor immunotherapy [82]. The main chain tends to adopt a helical conformation, and multiple chains can assemble into a triple helix through hydrogen bonding [83]. This triple-helix structure is generally associated with enhanced biological activity, particularly in facilitating recognition by pattern recognition receptors such as Dectin-1. Dectin-1, in particular, recognizes β-glucans and forms higher-order complexes with them and it synergizes with TLR2 and TLR4 to amplify cytokine production and inflammatory responses [84–86]. Polysaccharides containing β-(1 → 3)-glucosyl groups induce the production of cytokines such as TNF-α in monocytes and macrophages, consistent with the structure-receptor coupling described above [84].

Maca polysaccharides constitute a multi-component system comprising various structural types. Among the characterized fractions, α-glucans account for a relatively high proportion, while arabinogalactans (AG-I/AG-II) and pectin domains have also been identified [38, 39, 43]. The characterized maca α-glucans generally contain α-(1 → 4), α-(1 → 6), and α-(1 → 3) linkages, with branching predominantly at the O-6 position. This α-linkage-dominated structure contrasts markedly with the β-(1 → 3)/(1 → 6) backbone of classical immunoactive β-glucans that are recognized by Dectin-1 [85]. This raises a key question: How do α-linkage-dominant polysaccharides achieve immune regulation?

Receptor blockade experiments and cytological studies suggest that smaller maca polysaccharide fractions may participate more readily in signal transduction pathways [15, 45]. Dectin-1 is not the primary receptor for α-linked polysaccharides; however, synergistic interactions between different pattern recognition receptors, such as TLRs and C-type lectin receptors (CLRs), may compensate for this limitation [87]. Notably, receptor recognition typically requires matching between specific polysaccharide conformations and multivalent binding geometries. Glycosidic bond types indirectly modulate receptor interactions by shaping chain conformations and branching architectures, rather than directly determining receptor specificity [88]. For non-β-(1 → 3) glucan systems, a (1 → 6)-branched (1 → 4)-β-d-glucan has been shown to interact with TLR2 rather than Dectin-1, exerting anti-inflammatory effects by inhibiting TNF-α and IL-6 production [89]. α-(1 → 6)-Linked glucans can activate immune cells and induce cytokine production, as reported in studies of maca and related polysaccharides [39]. α-1,4-D-Glucan isolated from Tinospora cordifolia has been demonstrated to activate macrophages through TLR6 signaling, I-κBα degradation, NF-κB translocation, and subsequent TNF-α production [90, 91]. These findings are contingent on the polysaccharide source, substitution patterns, and aggregation state, necessitating validation in specific biological systems. In maca α-glucans, branching at the O-6 and O-3 positions has been identified in fractions such as MCP-1a, LMP-1, and MCP-3, contributing to their structural complexity [38, 44, 92]. Such branching enhances three-dimensional structural complexity and increases the density of potential receptor contact sites, providing a geometric basis for multivalent recognition.

### The decisive role of spatial conformation

Spatial conformation, as the ultimate manifestation of structural parameters, critically influences the recognition and binding of polysaccharides to immune receptors. Several mechanisms underlie the association between triple-helix conformation and enhanced immune activity [93, 94]. First, geometric matching: the regular grooves on the triple-helix surface facilitate spatial recognition by receptors such as Dectin-1, which undergoes ligand-induced oligomerization. Second, entropic advantage: pre-formed ordered conformations reduce conformational entropy loss during binding. Third, multivalent binding: the triple helix provides a regular arrangement of multiple binding sites, enabling cooperative receptor engagement. Fourth, slow dissociation kinetics: dissociation from ordered structures requires overcoming larger energy barriers, potentially prolonging receptor occupancy and signal duration.

The relationship between triple-helix conformation and biological activity has been confirmed across diverse polysaccharide systems. Triple-helical β-glucan from Lentinula edodes with moderate molecular weight (5.0 × 10^5^–1.5 × 10^6^ Da) exhibits significant antitumor activity, which may be attributed to the rod-like structure that enhances chain rigidity and improves receptor binding efficiency [95, 96]. The antitumor activity of schizophyllan correlates with the molar ratio of triple-helix to single-strand conformations in solution [97]. Samples with Mw exceeding 9 × 10^4^ Da predominantly exist as triple helices and exhibit strong antitumor effects. Studies on various polysaccharides have demonstrated that triple-helix conformations can activate immune signaling pathways through pattern recognition receptors, with activity modulated by both helical structure and surface functional groups [83].

However, triple-helix formation is governed by multiple structural factors. Generally, β-(1 → 3)-linked glucans with molecular weights exceeding 90 kDa are more likely to form triple-helix conformations, whereas increasing β-(1 → 6) branching loosens the triple-helix structure [98, 99]. Triple-helical lentinan exhibits optimal antitumor activity at intermediate molecular weights with diminished efficacy at both very low and extremely high molecular weights [100–102]. In Auricularia auricula (black fungus), high-molecular-weight β-glucan predominantly adopts single-helix or triple-helix conformations, whereas low-molecular-weight fractions tend to form random coils [96, 103].

Research on the chain conformation of maca polysaccharides remains in its early stages. Conformational data for most maca polysaccharide fractions remain unavailable, with the exception of the putative triple-helix conformation of MC-2 (indicated by Congo red assay) and the intermediate state of MP21 between spherical and random coil conformations [45]. Importantly, polysaccharide conformation is not static but represents a dynamic equilibrium modulated by solution conditions (pH, ionic strength, temperature), concentration, and intermolecular interactions [60, 104]. Triple-helix structures can denature into single strands at elevated temperatures or extreme pH, and may refold under favorable conditions. A study on ginger polysaccharides demonstrated that reducing the molecular weight from 1.1 × 10^6^ to 3.1 × 10^5^ Da caused the chain conformation to transition from rigid rods to semi-rigid chains and ultimately to random coils.[105]. Ginger polysaccharides modified at 130 °C exhibited optimal immunomodulatory activity, as evidenced by enhanced macrophage proliferation and elevated secretion of NO, IL-6, TNF-α, and IL-1β. These findings underscore that chain conformations measured in vitro may not fully represent those adopted under physiological conditions in vivo, warranting further investigation.

Collectively, the structure–activity relationships of maca polysaccharides are governed by the interplay of molecular weight, monosaccharide composition, glycosidic linkage patterns, branching degree, and spatial conformation. Future research should prioritize obtaining structurally defined single components and integrating multi-dimensional characterization techniques to establish definitive structure–function correlations.

## Conclusions

Maca polysaccharides represent a structurally diverse family of bioactive macromolecules, with molecular weights spanning three orders of magnitude (3.0–1951.0 kDa) and encompassing α-glucans, arabinogalactans, homogalacturonans, and mannose-containing heteropolysaccharides. This review establishes that their biological activities—including immunomodulation, antioxidant protection, anti-fatigue effects, and hepatoprotection—are governed by the interplay of multiple structural parameters. Structure–activity analysis reveals that medium-molecular-weight fractions (200–400 kDa) exhibit optimal immunomodulatory potency, while specific monosaccharide residues (mannose, galacturonic acid) and higher-order conformations (notably the putative triple-helix conformation of MC-2 suggested by Congo red assay) contribute to receptor recognition and signal transduction through TLR2/4-NF-κB, Nrf2/Keap1, and MAPK pathways.

Despite significant progress in structural characterization, critical challenges remain. The inherent heterogeneity of maca polysaccharides limits the availability of structurally defined fractions for mechanistic studies. Furthermore, pharmacokinetic profiles, intestinal absorption mechanisms, and structure-dependent bioavailability remain largely unexplored. Addressing these gaps will require integrated approaches combining advanced structural analytics (high-field nuclear magnetic resonance [NMR], mass spectrometry [MS] fragmentation, small-angle X-ray scattering [SAXS]), multi-omics technologies, and well-designed pharmacokinetic studies. Clinical translation necessitates standardized extraction protocols, quality control markers, and rigorous human trials.

The structural diversity and multi-target bioactivities of maca polysaccharides position them as promising candidates for functional food development. Their potential therapeutic applications remain to be validated through rigorous clinical investigation. The analytical framework and structure–activity principles established here may inform future investigations of polysaccharides from other Andean or Brassicaceae species, contributing to the broader goal of evidence-based development of plant polysaccharide therapeutics.

Looking forward, several research priorities warrant attention. First, the development of standardized extraction protocols and quality control markers is essential for ensuring batch-to-batch consistency. Second, pharmacokinetic studies are needed to understand the absorption, distribution, and metabolism of maca polysaccharides in vivo. Third, well-designed clinical trials are required to validate the health benefits observed in preclinical studies. To date, no clinical trials specifically evaluating purified maca polysaccharides have been registered or published. While several clinical studies have investigated whole maca preparations for endpoints such as sexual function and physical performance, attribution of observed effects to polysaccharide components is not possible given the complex composition of these preparations. Finally, bridging the gap between laboratory research and industrial application remains a significant challenge that requires interdisciplinary collaboration. Addressing these priorities will accelerate the development of maca polysaccharides as functional food ingredients and inform future clinical evaluation of their therapeutic potential.

## Data Availability

No new data were created or analyzed in this study. Data sharing is not applicable to this article.
